# Volition and control in law and in brain science: neurolegal translation of a foundational concept

**DOI:** 10.3389/fnhum.2024.1401895

**Published:** 2024-09-03

**Authors:** Julia F. Christensen, Caroline Rödiger, Lisa Claydon, Patrick Haggard

**Affiliations:** ^1^Department of Cognitive Neuropsychology, Max Planck Institute for Empirical Aesthetics, Frankfurt/M, Germany; ^2^School of Law, University of Manchester, Manchester, United Kingdom; ^3^School of Law, Open University, Milton Keynes, United Kingdom; ^4^Institute of Cognitive Neuroscience, University College London, London, United Kingdom; ^5^School of Advanced Study, Institute of Philosophy, University of London, London, United Kingdom

**Keywords:** volition, action control, human, law, action, loss of control, fear, anger

## Abstract

The law assumes that healthy adults are generally responsible for their actions and have the ability to control their behavior based on rational and moral principles. This contrasts with some recent neuroscientific accounts of action control. Nevertheless, both law and neuroscience acknowledge that strong emotions including fear and anger may “trigger” loss of normal voluntary control over action. Thus, “Loss of Control” is a partial defense for murder under English law, paralleling similar defenses in other legal systems. Here we consider the neuroscientific evidence for such legal classifications of responsibility, particularly focussing on how emotional states modulate voluntary motor control and sense of agency. First, we investigate whether neuroscience could contribute an evidence-base for law in this area. Second, we consider the societal impact of some areas where legal thinking regarding responsibility for action diverges from neuroscientific evidence: should we be guided by normative legal traditions, or by modern understanding of brain functions? In addressing these objectives, we propose a translation exercise between neuroscientific and legal terms, which may assist future interdisciplinary research.

## Volition and control in law and in brain science: a neurolegal translation exercise

All known human societies have some concept of responsibility for action. Healthy adults are held responsible for actions that they voluntarily choose and execute, but they are not generally responsible for actions that are involuntary. In **common law**^1^ systems, criminal responsibility requires *mens rea*: the agent must have intended the criminal action. This concept recalls psychological theories of action in which individuals are capable of appropriate and rational action because they plan their actions on the basis of goals and outcomes (Fishbein and Ajzen, 1975; Ajzen and Fishbein, 1980; Ajzen, 1985). Importantly, the law recognizes that not all human actions conform to this model. Recent psychological and neuroscientific studies likewise agree that the capacity of human volition is both profoundly limited (Libet et al., 1983; Wegner, 2003), and also routinely overestimated. For example, Libet argued that the timing of subjective intention came too late to provide active control of action generation, and was limited, at best, to a form of veto. Similarly, self-serving biases (Bandura, 1984, 2002) and misperceptions about the sources of our own actions may lead people to overestimate their own volitional agency.

In fact, human behaviors rarely have simple causes. Most action choices reflect complex interplays between neurocognitive factors within an individual on the one hand, and social/environmental processes on the other. Nevertheless, the criminal law must judge individual **actions** in a reasonable and consistent way, including classifying them as voluntary or not. Here we consider how this legal classification relates to neurocognitive concepts of the initiation, control, and awareness of actions. We focus on the partial defenses of loss of control and of diminished responsibility in England and Wales, though the points we raise may also be relevant to many other jurisdictions where similar principles apply. English law generally assumes that healthy adults are responsible for their actions, because they have the ability to control their behavior based on rational and moral principles (Moore, 2020). This view of action differs from more mechanistic views found in recent neuroscientific research. That research often stresses specific brain subsystems that drive behavior. For example, actions can arise from habit (Graybiel, 2008), or from specific calculations for reward maximization (Talmi et al., 2009). Some “neurolaw” approaches emphasize neural correlates of individual *traits* (Philippi et al., 2015), or the relation between stable measures of individuals’ brain function and their offending behavior (Kiehl et al., 2018), in addition to the standard emphasis on actions as individual events (Morse, 2004, 2023). Such general traits are assumed to contribute to the generation of particular culpable actions. Here, we take a complementary *event-based* approach, by focussing on actions themselves. The relation between specific actions and stable enduring traits of the agent is outside our scope.

**Figure 1:**
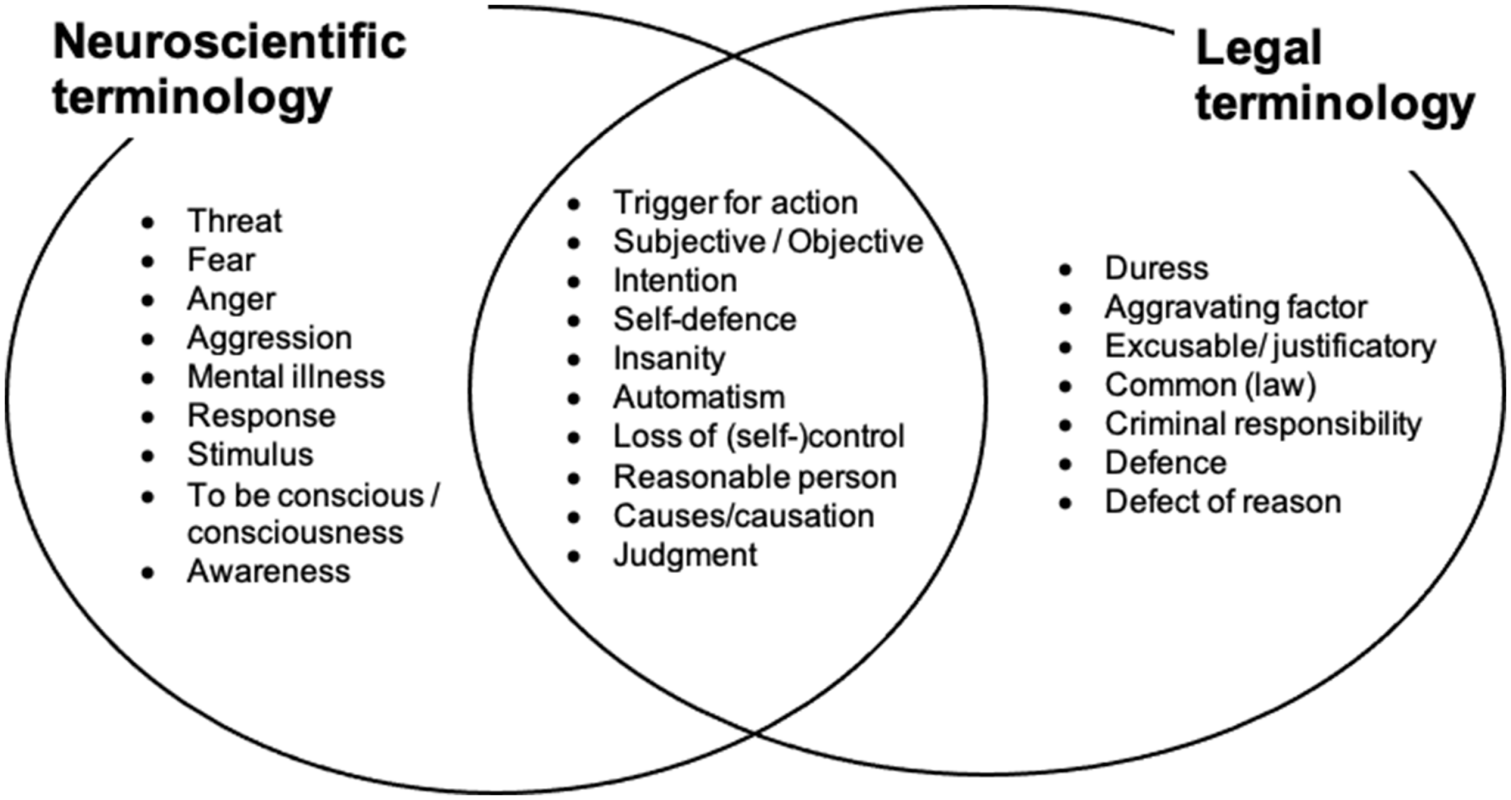
Key terminology for neuroscience and law. Each discipline has specific vocabulary that differs from the definition of these words in everyday language. Some terms are shared between the two disciplines, but each discipline has their own definition of those terms. Friction in interdisciplinary teams can occur when terms are not used in the discipline-specific way, or, when scholars are unaware of the presence of specific definitions of terms within the “other” discipline. The left column lists concepts that are relevant to responsibility and have a technical meaning within neuroscience, but which lack a corresponding technical definition in law, and would therefore fall back to the meaning based on everyday usage, potentially leading to incommensurability. The right column lists concepts that have a technical meaning in law, but which create difficulties in neuroscience because they cannot be readily related to a brain circuit or cognitive process, and so fall back to meaning based on everyday usage. The central, intersection column lists key terms that are important for both the neuroscience of responsibility and for legal responsibility, but which have a meaning restricted to just one discipline, or have distinctly different meanings within each discipline. Table 1 part C clarifies these concepts by directly contrasting neuroscientific and legal definitions.

Specifically, we consider the neural mechanisms that may underlie a specific action that constitutes a crime. We then try to relate these mechanisms to the legal treatment of the same action. In particular, we ask how a scientific understanding of the neural mechanisms involved in causing a specific action should influence the legal treatment of the action. We develop this approach in the context of Loss of Control defenses. Figure 1 and Table 1 show the different definitions and terminology that have been used in neuroscience and in law in discussing responsibility for actions.

**Table 1:** Conjunction and disjunction of terminologies relevant to loss of control.

Part A: neuroscientific terminology relevant to legal loss of control	
Threat	Threats trigger specific **automatic** physiological and behavioral reactions, controlled by low-level brain stem and amygdala circuits. Threats lead to a psychological state of fear. Threats may be physical or psychological in nature. Types of physical threat include threats to bodily integrity (damage to physical body, death). Types of psychological threat include verbal threat of physical control, physical aggression, psychological ‘mind’ control, and behavioral variants of these. An additional category of **‘social threat’** has been suggested. Social threat does **not trigger automatic responses**. It is controlled by brain systems involved in reasoning, decision-making and self-control (e.g., the dorsolateral prefrontal cortex). The psychological state associated with social threat is anger, rather than fear. Types of social threat include threats to self-esteem, threats to culturally defined social relations, status, etc.
Fear	Psychological and physiological state elicited by a physical or psychological threat. Neurophysiological manifestations of fear depend on the level of threat severity, and include withdrawal, aggression, and freezing (often called fight or flight response). Some degree of cognitive control of fear responses is possible, through conscious appraisal and learned cognitive strategies. However, imminent and severe threats trigger fear reactions that cannot be voluntarily controlled.
Anger	Psychological and physiological state elicited by a social threat, typically from another individual. Anger responses to a threat involve frontal brain structures implied in self-control, reasoning and decision making. Behavioral correlates of anger include withdrawal or aggression.
Aggression	Threatening or potentially threatening behaviors emitted by specific neural circuits, often in the context of fear or anger, and often triggered by specific stressor events.
Mental illness	Mental illnesses are health conditions involving changes in emotion, thinking or behavior, or a combination of these. Clinical manuals (e.g., DSM-V) give diagnostic criteria for different types of mental illness. Current clinical approaches link specific patterns of change to altered function of specific neural substrates (e.g., the National Institutes of Health Research Domain Criteria approach).
Response	In psychology, a response is any event caused by a stimulus. Responses can be behavioral, verbal, physiological, hormonal, neural, etc.
Stimulus	Any physical event in the internal or external milieu of an organism that is registered by the organism’s sensory systems, and potentially leads to a response.
Consciousness	Neurological state implying full possession of mental capacities, and depending on integrated brain activity. The specific patterns of brain activity responsible for consciousness remain controversial. Reductions or alterations in brain activity are associated with reduced levels of consciousness, such as vegetative states.
Awareness	Neurocognitive state of experienceing one’s own bodily states and behavior. Some bodily responses to threat, for example, may occur without awareness.

Part B: legal terminology relevant to neurobiology of behavioral control (english legal terminology)	
Duress	**Duress** by **threats** and **duress** by **circumstances** are both defenses developed at common law. To provide a **defense**, the threat must be made by the person who has instructed the accused to commit the offense. **Duress** by **threats** consists of three elements: (1) the accused must have reasonably believed that she or someone for whom she reasonably felt responsible was **threatened** with death or serious injury unless she committed the offense and (2) a sober person of reasonable firmness in those **circumstances** would have reacted as she did. (3) However, **duress** will be excluded in relation to murder, attempted murder, and does not apply in circumstances where the accused is involved in criminal activity, or she could reasonably have taken steps to avoid the threat. **Duress** by **circumstances** arises where the **threat** comes from the defendant’s **circumstances** rather than explicitly from a person. For example, in *Willer* (1986) 83 Cr App R 225, the defendant drove his car onto a pavement in order to escape from a gang of youths who intended to do serious harm to him and his passenger.
Aggravating/Mitigating factor(s)	An aggravating factor makes a crime more serious and might raise the sentence. A mitigating factor makes a crime less serious and might reduce the sentence. A list of aggravating and mitigating factors that may be relevant to the offense of murder is laid down in Schedule 21 of the Criminal Justice Act 2020.
Excusable/ justificatory	Early ideas of responsibility assumed that in certain circumstances criminal acts could be **justified**. One example might be acting in self defense. In such cases the justification rendered the use of force lawful. Most offenses of serious violence use the word unlawful as part of the offense definition, e.g., murder requires an unlawful killing. An **excusing** condition may totally relieve criminal responsibility as in the case of the general defense of insanity, or may diminish the culpability of those who are criminally responsible, and result in reduced punishment. Loss of Control and Diminished Responsibility are examples of partial defenses to a specific crime, namely murder. They are unusual in the criminal law in that they partially remove responsibility for the crime and lessen criminal culpability. Diminished Responsibility partially excuses responsibility. However, whether loss of control should be considered a justifying condition, or an excusing condition, remains unclear, since loss of control involves some elements of each. These partial defenses allow judges flexibility in sentencing which they would not otherwise have as murder carries a mandatory life sentence.
Common law	A system of laws which is based on customs and court decisions.
Criminal responsibility	Refers to the responsibility for the criminal act. The focus is on the moment of the crime. Subjective elements are relevant in assessing responsibility for the manner in which the crime is carried out. Intentional, reckless and negligent actions may all incur criminal responsibility. These designations are ways of denoting descending degrees of criminal responsibility, with intention being the most culpable mental state. The law recognizes factors that may reduce culpability. For example, the two partial defenses to murder (diminished responsibility and loss of control) introduce excusing conditions based on what the law views as relevant mental states that may partially reduce culpability. An assessment of whether the defendant’s action in killing was intentional will be required to establish that the killing was murder. Note that **intention** in the criminal law carries a different meaning to that used normally in philosophy or in psychology and neuroscience – see “intention” further down in this table. In the offense of murder it refers to an intention to kill or cause serious bodily harm and may be found by the jury in circumstances where death was a virtually certain result of the accused’s actions and the accused knew that death was virtually certain to result from her actions. *R v Woollin* [1998] UKHL 28.
Defense	The law distinguishes between general and partial **defenses**. General **defenses** such as **self-defense** provide a complete defense to any criminal charge, whereas partial **defenses** such as **diminished responsibility** or the **loss of control** defense reduce the defendant’s liability from murder to manslaughter.
Defect of reason	Insanity requires that the defendant’s legally defined **disease of the mind** must have caused a **defect of reason**, not caused by intoxication, or a brief psychotic episode; R v Coley [2013], EWCA Crim 223. This definition will exclude psychiatric conditions where the accused knew that what they were doing was legally wrong, but because of volitional impairment cannot refrain from acting. The terminology originates with the M’Naghten Rules (1843) 10 Cl & Fin 200, All ER [1843–60] 229.

Part C: terms with different meanings in neuroscience and in law		
	Neuroscience	Law
Trigger	A ‘trigger’ is a ‘stimulus’. Stimuli trigger behaviors. A stimulus that triggers a behavior can be extrinsic (e.g., I see a lion – I run away), or intrinsic (e.g., I’m scared – I run away), or a mix of both. Neuroscientific research shows that individuals may not be aware of all stimuli that influence their behavior	An example of the use of a “qualifying trigger” is set out in section 55 of the Coroners and Justice Act (2009). The defense of loss of control, a partial defense to murder, is not available to those acting for revenge (section 54 of the act). To establish the defense, the **loss of control** must have a **“qualifying trigger”**. Triggers are defined in section 55 of the Act and must have been caused by one of two things. (1) D (the **D**efendant) must have feared serious violence from the victim (“fear **trigger**”) OR been triggered by (2) a thing or things done or said (or both) of an extremely grave character, that caused D to have a justifiable sense of being seriously wronged. There is a further requirement as to the effect of the “trigger” imposed by section 54 “a person of D’s sex and age, with a normal degree of tolerance and self-restraint and in the circumstances of D, might have reacted in the same or in a similar way to D.”
Subjective / Objective	The term ‘subjective’ refers to an individual’s experience. The content of that experience can be reported, e.g., verbally, or in writing. Scientific studies typically investigate the relation between subjective states and objective measures or events (e.g., stimuli, behaviors). ‘Objective evidence’ is non-experiential, and directly measurable. Objective evidence in neuroscience includes behavior, neuroimaging and neurophysiological data (e.g., fMRI, EEG), neuropsychological data, and implicit behavioral data.	A ‘subjective’ element describes a behavior or situation as it was perceived by the accused, whereas an ‘objective’ element is based on asking whether a reasonable neutral observer would have acted, or perceived a situation, in the same or in a similar way to the accused. Typically this ‘objective’ role is adopted by the jury and based on a mix of a “common sense” understanding of human psychology, advice from the judge on the law, and its application to the case; plus their own life experience. Thus, the terms ‘subjective’ and ‘objective’ in law seem equivalent to ‘self’ and ‘others’ in neuroscience.
Intention	In neuroscience, intentional actions are a class of action that address to the goals and motivation of the agent, as opposed to automatic, reflexive or habitual actions. Intentional actions typically involve preparatory activity in the agent’s brain, notably the frontal and prefrontal cortices. A sense of agency is experienced with respect to the outcome of intentional actions, but not with respect to similar events occurring in the absence of intention. Sense of agency is reduced under coercion, and during fear/anger (Haggard et al., 2002; Yoshie and Haggard, 2013; Christensen et al., 2016).	**Intention** is the most serious level of mens rea (guilty mind), which describes the greatest culpability and blameworthiness. It refers to a defined mental attitude in the case of murder – an intention to kill or cause serious bodily harm and may be found by the jury in circumstances where death was a virtually certain result of the accused’s actions and the accused knew that death was virtually certain to result from her actions. *R v Woollin* [1998] UKHL 28.
Self-defense	Behavioral reaction in response to physical and psychological threat. Accompanied by specific and unspecific physiological reactions, fight or flight response. Aggression out of a fear response (“fight response”) occurs if alternative responses involving withdrawal (“flight response”) are not available.	**Self-defense** is a general **defense** to any charge where the defendant uses force to prevent a crime. It provides a defense where the defendant uses force to protect himself, his property or another person from harm. The public **defense** provided by section 3 of the Criminal Law Act (1967), provides a defense where the defendant uses force to prevent a crime or in effecting or assisting lawful arrest. Section 76 of the Criminal Justice Act (2008) clarifies the test to be applied to ascertain, whether the reaction is reasonable. Firstly, there is a triggering condition – belief in the circumstances that render force necessary – tested by a reasonableness test. Genuineness of belief is key as is degree of force used in terms of response to threat.
Insanity	Term historically used for ‘mental illness’ or ‘psychopathology’, and not in current use. Guidance by UK Department for Work & Pensions, Office for Disability Issues notes: *The word “insane” is to be avoided (along with mental patient, mad). It is recommended to use “person with a mental health condition”.*	**Insanity** as a **defense** has three requirements as set out in the *M’Naghten Rules* (1843): to establish the defense at the time of the criminal act, D must be “laboring” under a **defect of reason** from a legally recognized **disease of the mind** and raise evidence that their conduct fell within one of two cognitive limbs of the rules: either (1) D did not know the nature or quality of his act, or (2) he did not know that it was legally wrong. From a legal point of view, a tight control is kept on what is recognized as a disease of the mind although in practice many legal cases will involve a medically recognized condition (Mackay et al., 2006). The law focusses on the whether the disease of the mind results from an internal or an external factor. Most cases will be deemed to have an internal cause such as epilepsy *Sullivan* (1984) AC 156. Such cases fall within the category of **disease of the mind**. This legal definition often excludes claims of insanity, where D lacks volitional control, even though brain science describes several disorders that affect volitional control. Crucially, a person with a disorder of volitional control may know the nature and quality of her act and that it is wrong, but may nevertheless perform the act.
Automatism	Automatic responses without the agent’s intention (e.g., reflex movements in response to a stimulus; startle responses to unexpected salient events; habitual responses to a context). The agent may or may not be aware of the response.	The **defense** of “sane **automatism**” is raised when some external or purely physical factor affected D’s conscious control of her body at the time of the crime. D’s conduct is regarded as insane automatism if it arises from an internal factor or disease and is therefore likely to recur, e.g., *Burgess* [1991] 1 WLR 1206. In Burgess, D was charged with a violent assault. Burgess claimed he was sleepwalking. The court deemed he was in a dissociative state, this was an internal cause and, therefore, insane automatism was the appropriate defense.
Loss of control	Several neurological conditions are associated with involuntary movements (e.g., tic disorders; tremors). ‘Loss of (self-)control’ often refers to situations where the agent appears to be unable to stop specific patterns of action, even when these are maladaptive (e.g., obsessive-compulsive disorders, eating disorders, impulse control disorders, frontotemporal dementia).	Refers to a variety of matters that may lessen culpabitity. See ‘**Loss of control** as a partial **defense** to murder’, discussed under ‘**trigger**’ above. See also ‘**insanity**’ or ‘**automatism**’ where loss of cognitive control may excuse from culpability. Further, see ‘**self defense**’, and ‘**diminished responsibility**’. In law, there is no one concept of self-control, but rather a number of circumstances that may excuse. The law imposes restrictions to limit the availability of the defense ‘**Loss of control**’ – e.g., in **duress**, the claim is restricted by the limitations on those who can access the defense (see above). For example, people who behave impulsively may be seen as “dangerous” by the law and possibly requiring confinement. *Bratty v Attorney General for NI* [1963] AC 386.
Reasonable person	Neuroscience recognizes individual differences in thought and behavior, that have both genetic and experiential bases. There are no objective neuroscientific measures, nor thresholds that might describe what a ‘normal degree of tolerance and self-restraint’ might be (= ‘reasonable person test’ in law). Current methods such as neuroimaging do not allow reliable out-of-sample predictions about such person-level characteristics.	The term ‘reasonable person’ is used in law to allow ‘objective’ judgment by the jury of D’s behavior. It has variable meanings dependent on specific offenses and defenses. Within the context of the **loss of control defense**, the ‘**reasonable person**’ refers to a person of D’s sex and age, with a normal degree of tolerance and self-restraint and in the **circumstances** of D, might have reacted in the same, or in a similar way to D (section 54 (1) (c) of the Coroners and Justice Act 2009).
Causes/causation	Neuroscience uses the concepts of instrumental action, or operant action, to describe the relation between an action and the outcome caused by that action. A sense of agency typically results from causing the outcome through one’s intentional action.	When assessing liability for a “result crime” such as homicide offenses, it is essential to demonstrate a **causal** link between the defendant’s conduct (e.g., squeezing the trigger on the pistol) and the result (a person’s death). If the defendant’s conduct did not *cause* the result, he will not be liable for the crime. This is called “but for” causation. The concept of legal causation also applies. It measures the culpability for the act. In practice this means culpability in the form of a connection between the fault and the resulting act. How this is established varies from offense to offense. Thus, in dangerous driving cases the manner in which the car is driven will be a measure of the fault of D. If the driving is faultless, then liability will not attach to D for a fatal crash *Taylor [2016] UKSC 5.*
Judgment	A psychological subject makes judgement by applying predicates to events or entities. Thus, one can judge whether one caused an event or not (judgment of agency); one can judge the praise/blame-worthiness of a person, or of an action (as in a “trolley problem”).	Judgment (legal spelling) is the decision of a court. D’s use of judgment will also affect assements of culpability. For example, in **self defense**, judgment as to the reasonableness of the force used is important to the success of the defense.

Section A explains key neuroscientific terms relevant to loss of control. Section B explains key legal terms relevant to loss of control as a partial defense for murder. Finally, section C identifies terms that are used in different ways, and potentially have different meanings, in neuroscientific vs. legal treatments of agency, responsibility and control.

The law recognizes that certain environmental situations can provide ‘**qualifying triggers**’. These situations may lead directly, and presumably unavoidably, to action, and lie outside of an agent’s normal voluntary control. Neuroscientifically speaking, this would imply a basic neuromotor mechanism intruding into the sphere of rational, responsible action. The ‘loss of control’ defense is a legal defense arguing for reduced responsibility, on the grounds that one of these qualifying triggers is present. Actions performed out of fear or out of anger provide the most ready examples of the ‘loss of control’ defense. In particular, when an action is performed in situations of fear or anger, criminal liability for a resulting death may be reduced from murder to manslaughter. The crucial difference between murder and voluntary manslaughter is the intent to kill. The partial defenses provide for a reduction in culpability where D (the defendant) intends to kill, but there is an explanation for the behavior that may reduce legal culpability. In the “partial defenses” (a legal technical term that refers to this particular type of defense), the criminal law is concerned primarily with the nature and the severity of those causes of the action that derive from the situation (i.e., the factors that lead to the agent being afraid or angry). Therefore, the law seems to assume that agents can be less intentional when they are strongly afraid, or strongly angry. In principle, neuroscientific knowledge could either support this assumption or call it into question.

In section 1, we outline a neurocognitive classification of human behavior, focussing on behaviors that are legally relevant. We explore whether the complementary legal and neuroscientific approaches to human behavior should be seen as compatible but alternative conceptual schemes that merely require some interdisciplinary translation, or whether they rather involve fundamentally incommensurable ways of thinking about the same events. In section 2, we consider the societal impact of the areas where neuroscience and law disagree, and we use these as a blueprint for future interdisciplinary research.

## Section 1: neuroscience on the origins of human behaviors

A legal plea of ‘Not guilty’ is a personal statement about agency: ‘I did not do it’. There are at least 4 factors that could motivate this claim.

1. The agent in question truly did not do it.
2. The claim reflects secondary gain considerations (e.g., the agent did it, and knows that they did it, but they are lying).
3. The agent did it, but they do not experience that they did it, because some neurocognitive mechanism reduced their ability to control their action, and/or their experience of agency with respect to the action (Haggard et al., 2002; Yoshie and Haggard, 2013; Christensen et al., 2016). In this case, the agent’s claim ‘I did not do it’ may be coherent from their first-person perspective.
4. The agent did it, but either does not or did not realize it was legally wrong.

These four situations attract very different attention from the law, although they may not separate very clearly in any particular case. Broadly speaking, however, if scenario 1 can be established, the agent is clearly not criminally liable. If scenario 2 can be established, the agent is criminally liable. Scenario 4 is also recognized as a “mistake of law,” although there is a general assumption that agents should have some knowledge, simply through being members of society, about what actions are proscribed and what are allowed (Matthews, 1983). The most interesting situation for our current neurolaw discussion is situation 3, since it speaks directly to the loss of control defense. The law is rightly skeptical regarding situation 3, because claiming situation 3 is very attractive when situation 2 actually applies. Consider an agent who knew at the time what they were doing, did it nevertheless, and still knows now that they have done it. This agent has a strong motivation to claim that their action was, in fact, beyond their voluntary control. Clearly, any claim of situation 3 needs to present convincing evidence that the agent did not have a conscious experience of willing and controlling the action. Here we consider whether neuroscientific evidence can potentially clarify the circumstances under which situation 3 is likely, and also when it is unlikely. We do so in the context of one particular legal argument, namely loss of control (see Table 2). The partial **defense** to murder of **loss of control** is laid down in sections 54 and 55 of the *Coroners and Justice Act 2009.*^2^ We will not consider situation 4.

**Table 2:** Extract of the coroners and justice act 2009, section 54.

**54. Partial defense to murder: loss of control** Where a person (‘D’) kills or is a party to the killing of another (‘V’), D is not to be convicted of murder if– D’s acts and omissions in doing or being a party to the killing resulted from D’s loss of self-control, the loss of self-control had a qualifying trigger, and a person of D’s sex and age, with a normal degree of tolerance and self-restraint and in the circumstances of D, might have reacted in the same or in a similar way to D. For the purposes of subsection (1)(a), it does not matter whether or not the loss of control was sudden. In subsection (1)(c) the reference to ‘the circumstances of D’ is a reference to all of D’s circumstances other than those whose only relevance to D’s conduct is that they bear on D’s general capacity for tolerance or self-restraint. Subsection (1) does not apply if, in doing or being a party to the killing, D acted in a considered desire for revenge. On a charge of murder, if sufficient evidence is adduced to raise an issue with respect to the defense under subsection (1), the jury must assume that the defense is satisfied unless the prosecution proves beyond reasonable doubt that it is not. For the purposes of subsection (5), sufficient evidence is adduced to raise an issue with respect to the defense if evidence is adduced on which, in the opinion of the trial judge, a jury, properly directed, could reasonably conclude that the defense might apply. A person who, but for this section, would be liable to be convicted of murder is liable instead to be convicted of manslaughter. The fact that one party to a killing is by virtue of this section not liable to be convicted of murder does not affect the question whether the killing amounted to murder in the case of any other party to it. **55. Meaning of ‘qualifying trigger’** This section applies for the purposes of section 54. A loss of self-control had a qualifying trigger if subsection (3), (4) or (5) applies. This subsection applies if D’s loss of self-control was attributable to D’s fear of serious violence from V against D or another identified person. This subsection applies if D’s loss of self-control was attributable to a thing or things done or said (or both) which— constituted circumstances of an extremely grave character, and caused D to have a justifiable sense of being seriously wronged. This subsection applies if D’s loss of self-control was attributable to a combination of the matters mentioned in subsections (3) and (4). In determining whether a loss of self-control had a qualifying trigger— D’s fear of serious violence is to be disregarded to the extent that it was caused by a thing which D incited to be done or said for the purpose of providing an excuse to use violence; a sense of being seriously wronged by a thing done or said is not justifiable if D incited the thing to be done or said for the purpose of providing an excuse to use violence; the fact that a thing done or said constituted sexual infidelity is to be disregarded. In this section references to ‘D’ and ‘V’ are to be construed in accordance with section 54.

Table sets out the requirements for the partial defense of loss of control partial defense to murder (Coroners and Justice Act 2009, section 54).

The loss of control defense repeals the previous common law partial defense of provocation and applies in cases where a person claims to have lost self-control [section 54(1)(a)] if this conduct was not motivated by a desire for revenge [section 54(4)]. Furthermore, it must have been caused by a so-called **qualifying trigger**, i.e., by a **fear** of serious violence [section 55(3), ‘**fear**’ trigger] and/or by a thing or things done or said (or both) which constituted circumstances of an extremely grave character, which caused the person to have a justifiable sense of being seriously wronged [section 55(4), ‘**anger**’ trigger: we take anger as the neurocognitive emotional state that results from being ‘wronged’]. However, the defendant cannot rely on one or both of the triggers if they themselves incited the trigger situation as an excuse to use violence [section 55(6)(a) and (b)], e.g., if A taunts B to make B angry, A could not then claim that fear of B’s serious violence is a qualifying trigger. In addition, section 55(6)(c) provides another important exclusion from the ‘**anger**’ trigger: it excludes things said or done constituting ‘sexual infidelity’. Sexual infidelity is, thus, not relevant to whether someone has been ‘seriously wronged’. The final requirement within the **loss of control defense** is a so-called ‘**objective** test’. The defendant’s reaction must have been objectively understandable in a way that a person of the defendant’s sex and age, with a normal degree of tolerance and self-restraint and in the **circumstances** of the defendant, might have reacted in the same or in a similar way [section 54(1)(c)]. Interestingly, this so-called ‘objective’ test in fact introduces a norm-related element to the defense: would it be ‘normal’ to behave in this way, given the situation? The mention of a person’s sex as part of this norm is particularly striking. On the one hand, it could be seen as biological determinism, or as an unwarranted, gendered bias. On the other hand, one legal text speculates that it reflects an odd legacy from case law, given that there is no evidence for different levels of normal tolerance or self-restraint between the sexes (Child and Ormerod, 2015). If all these **requirements** are met, the defense will reduce the defendant’s liability from murder to manslaughter. Importantly, this defense therefore avoids the mandatory life sentence for murder.

## Section 2: fear and anger for law – through the lens of neuroscience

From the neuroscientific perspective, there is something unusual in the way the law deals with criminal acts. Both law and neuroscience explicitly acknowledge two states that may lead to a **loss of normal voluntary control over action** (out of **fear**, vs. out of **anger**). However, the two disciplines use very different means to establish whether these states were present or not.

The first scientific assessments of fear and anger states were made by Cannon (1927, 1931) who postulated that visceral reactions in emotional states are unspecific (i.e., they do not show a specific pattern depending on the particular emotional state). Ax (1953) combined several psychophysiological measures of autonomous nervous system activity (e.g., heartrate, ECG; respiratory rate; galvanic skin response, GSR; electromyography, EMG, etc.) to assess the difference in psychophysiological arousal between fear and anger states. Ax made volunteer participants, in turn, fearful and angry. Anger caused higher diastolic blood pressure, higher EMG levels, and more frequent galvanic skin responses than fear. Conversely, when participants were in a fearful state, they showed an increased tonic baseline level of GSR, an increased number of phasic EMG peaks and a higher respiration rate. These differential effects were broadly replicated later by Schachter (1957).

However, the law has no *a posteriori* evidence about such neurophysiological states at the time of the act. Therefore, these differential neurobiological qualities are useful chiefly because they correlate with the different subjective experiences of fear and anger, respectively. In fact, fear and anger frequently co-occur in loss of control situations, but they have different effects on capacity for voluntary action, and are indeed treated differently in section 55 of the Coroners and Justice Act (2009), see Table 2.

Such differential diagnosis is borne out by neuroimaging studies. In a meta-analysis, Vytal and Hamann (2010) identified 13 clusters of brain activity, mainly located in left inferior frontal gyrus (IFG), as specific to anger states. Fear > Anger contrasts (comparisons) revealed 11 significant clusters, mainly located in left amygdala. Threat processing that leads to fear is confined to specific circuits, including the amygdala (Davis, 1994 LeDoux, 2003, 2012; Adolphs et al., 1994), while anger states recruit higher-level brain circuitries such as PFC. While it is true that no ‘distinct systems’ exist in the brain, and all systems are in some way connected with one another (Pessoa, 2017, 2023a,b; Pessoa and McMenamin, 2017), put simply, anger is a cortical response, and therefore participates in the wider cognitive systems for rational control of appropriate action. In contrast, fear is a subcortical response, linked to evolutionarily ancient systems for control of basic survival-oriented behaviors.

### Fear

**Figure 2:**
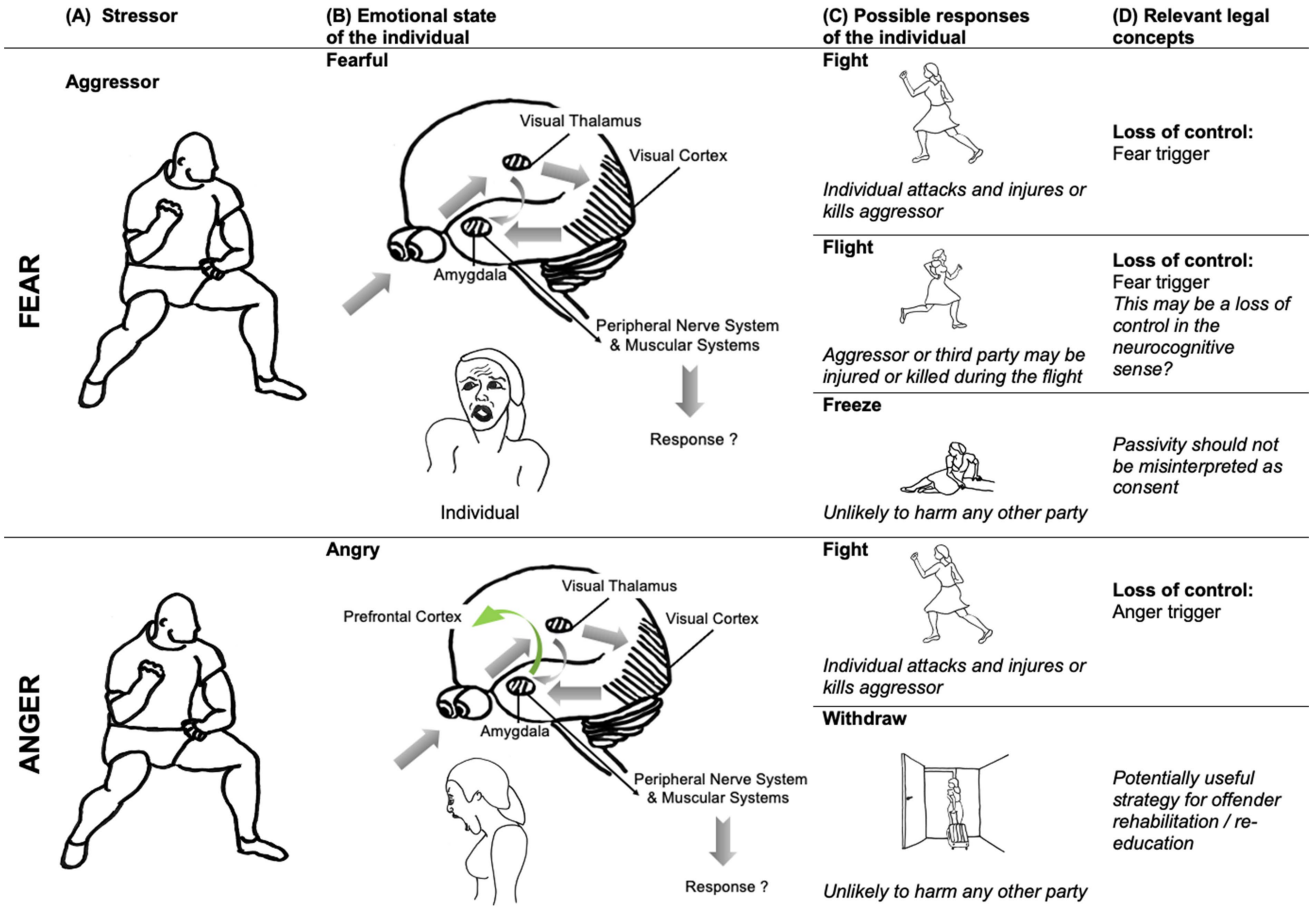
The neural fear/threat circuit and its legal implications. Colomn A: An aggressor is considered a stressor stimulus that is detected by an individual’s sensory systems. Column B: Visual information relating to the stressor is relayed via the visual thalamus to the visual cortex and directly to the central nucleus of the amygdala. The amygdala has direct projections with the peripheral nerve and muscular systems of the body, which prepare the body for appropriate response to the stressor, by activating specific and stereotyped defense behaviors. Depending on the sensory input, the individual’s appraisal will lead to different emotions: fear or anger. Column C: In fear contexts, three distinct response types are prominent: “fight, flight or freeze.” In anger contexts, reactive aggression responses predominate, but can be downregulated by the prefrontal cortex (PFC) in most circumstances, as when the individual withdraws. Column D: The individual’s response is legally significant: for example, the individual’s fight response may lead to injury, or even death, of the aggressor. The flight response might unintentionally harm a third party. Classically, legal considerations of loss of control apply to fight responses, and depend on identifying a fear or anger trigger. Neuroscientific evidence that multiple responses can arise from the same circuitry raises the question of how to treat these responses consistently in law. Illustration, text and neural models elaborated on basis of illustration from Ledoux (2003). Drawings: © Main Author anonymized.

Fear has been described as a ‘*physiological state that intervenes between stimuli and responses (…). Conscious fear can occur when conditions are favorable, but such conscious states come about through different processes that involve different [neural] circuits. The function of the neural circuit [of fear] …is to coordinate brain and body resources to increase the chance of surviving the encounter (…)*’ (Ledoux, 2014, p. 2873). Neuroscientific accounts of brain mechanisms in humans are often based on comparative research with other animals. In the case of fear, the crucial role of the amygdala circuit has been established in studies with rodents (LeDoux, 2003). Responses to threats are stereotyped and species-specific. Briefly, the central nucleus of the amygdala is connected via the periacqueductal gray with the brain’s motor systems, to activate specific defense responses (“fight, flight or freeze” responses). When danger is detected by a sensory system such as vision or hearing, the information is processed by the thalamus and relayed directly to the amygdala. This evaluation activates the autonomous nervous system (e.g., increasing heart rate and blood pressure), and the neuroendocrine system (e.g., triggering stress hormone release by action on the pituitary and adrenal glands). These changes prepare the organism for the optimal behavioral response to threat. The amygdala also directly impacts the motor systems to produce fight and flight behaviors by acting on the hypothalamic–pituitary–adrenal axis, releasing neurotransmitters implicated in motor control and thus provoking motor responses such as trembling. See Figure 2 for an illustration.

Human experimental studies are largely based on showing fearful or angry faces to healthy volunteers while recording their brain activity. Such threatening stimuli activate similar neural circuits in humans to those in animals (LeDoux, 2003; Denson et al., 2009b). The subcortical circuits underlying threat-processing and fear are thought to be evolutionarily-conserved. However, humans have a strong projection from the prefrontal cortex to the amygdala. This projection is thought to play an important role in learned control of fear, and is a focus for cognitive therapies in anxiety disorders.

The legal concept of loss of control seems to suggest that brain circuits for fear should influence brain circuits for voluntary action. Indeed, some scientific experiments have investigated the effects of fear on control of voluntary action in humans. Transcranial magnetic stimulation (TMS) applied over the motor cortex during the observation of fearful body positions results in lower motor excitability in hand muscles responsible for grasping (first dorsal interosseous), than during observation of happy and neutral body positions (Borgomaneri et al., 2015). Conversely, excitability increases when participants see angry bodily expressions (Hortensius et al., 2016). This suggests a visuo-motor route within the brain that could modulate the tendency to approach a stimulus, with the modulation operating in different directions in the case of fear and anger.

To our knowledge, however, no studies with human participants have investigated whether and how intense fear-states in the individual themselves might lead to a loss of voluntary action control: such experiments would quite probably be unethical. Experimental studies confirm that fear and anger indeed influence the *subjective experience* of voluntary action control, and particularly the ‘sense of agency’ (i.e., the subjective experience of controlling one’s own voluntary actions, and, through them, their consequences in the external world). Using an implicit measure of sense of agency, based on time perception (the “intentional binding” paradigm; Haggard et al., 2002; Haggard, 2017), a laboratory analog of “fear” was induced (by means of the threat-of-shock paradigm; Davis et al., 2010; Robinson et al., 2013). Then, participants were asked to estimate the interval between their action and the subsequent outcome. Their time estimation (intentional binding) in the condition of fear was compared to their performance in a neutral condition, with no emotion induction. Longer time estimates in the fear condition were taken to indicate a reduced sense of agency. Thus, fear apparently undermined participants’ subjective sense of control over their ations (Christensen et al., 2019, exp. 1&2). Conversely, other research has shown that positive emotion and arousal induction lead to an increase in the subjective sense of control (Wen et al., 2015; Minohara et al., 2016). Thus, the concept that fear can lead to loss of control comes from experimental human work on relatively weak fear-inducing stimuli. Conversely, strong evidence from animal research with non-human mammals suggests a cornered, frightened animal will nevertheless attack when possibilities of escape have been exhausted (Seligman, 1971; Pichon et al., 2012).

This argument has interesting wider implications. If neuroscience demonstrates a mechanism that directly and powerfully shapes human behavior, it seems logical that engagement of this mechanism must be relevant if it occurs in legal contexts. An interesting future challenge for neurolaw will involve considering whether a single, identified neural mechanism, in this case the fear/threat response circuit, demands consistent legal treatments across the different behaviors, and thus legal cases, in which it figures.

### Anger

The case of anger is less clear. Anger has been defined as *a* ‘*syndrome of relatively specific feelings, cognitions and physiological reactions linked associatively with an urge to injure some target*’ (Berkowitz and Harmon-Jones, 2004). However, no single, dedicated low-level circuit for anger, analogous to that for fear (LeDoux, 2012), has been identified in animals. Research with animals shows that aggression out of anger can be viewed as a set of several distinct neuromotor elements, rather than a unitary nervous state. These elements include a *defensive* aggression circuitry (self-protection against harm), *reproductive* aggression circuitry (against competing individuals) and a *predative* aggression circuitry (against a possible prey) (Chi and Flynn, 1971; LeDoux, 2012). Interestingly, these responses are highly species-specific, and are often triggered by the need to defend territory, mates, kin or resources from conspecifics (Brosnan and de Waal, 2003; Roma et al., 2006).

Studies with humans have struggled to produce convincing methods for eliciting genuine anger in experimental situations, again perhaps because ethical reasons discourage making volunteer participants truly angry (Anderson and Bushman, 1997). Instead, milder experimental paradigms have been used, based on inducing anger through frustration. In human social experiments, anger has been induced, for example, by social humiliation (Ritter and Eslea, 2005; Leary et al., 2006), by economic inequity (Beyer et al., 2015; Klimecki et al., 2018), or by posing an “impossible task” to participants (Buss, 1961; Taylor, 1967; Pawliczek et al., 2013). These studies show widespread neural activity throughout the brain associated with frustration/anger, including both cognitive and ‘limbic’ (emotional) neural systems (e.g., Denson et al., 2009b; Blair, 2012). In the aforementioned study on the effects of fear on participants’ sense of agency, also a lab analog of “anger” was induced in a different group of participants (using an “impossible task”-frustration paradigm; Buss, 1961; Taylor, 1967). Similar to the fear condition explained above, as compared to a neutral condition, participants’ subjective sense of control over their own actions was again reduced when angry (Christensen et al., 2019; exp. 3). All three of these experiments showed a significant mismatch between the objective facts of an action, and participants’ subjective sense of control over it, both when participants were fearful, or angry.

### Intention

In criminal law proceedings, one cannot retrospectively establish exactly what happened at the moment of a crime and whether ‘loss of control’ applies, so the factors relevant to a particular legal scenario can never be definitively found in the experimental literature. But law and neuroscience have rather different ways to deal with these knowledge gaps. The law is concerned with the mind-set of the person (*mens rea*) at the moment of the act (*actus reus*). The law assumes that people generally act with **intent**, while recognizing that intended actions may have unintended outcomes. In this context **intent** or **intention** has a very different meaning for law and neuroscience. Intention in the law typically refers to intended outcomes. The question is whether or not the person had *the intention to kill* at the time of the killing. In contrast, neuroscientists use the term intention or intentional to refer to the neural pathways that drive motor action, rather than the representation of the final outcome of the action. For example, intentional actions necessarily involve the cerebral cortex, and the final common path from the brain to the body through the primary motor cortex. This contrasts with non-intentional forms of action, such as reflexes, which may bypass the brain entirely. We elaborate on this disinction in Table 3.

**Table 3:** Overview of the neural circuitries involved in the emotional states of fear and anger.

	Fear	Anger
Definition	Fear is *a nonsubjective physiological state that intervenes between stimuli and responses (…). Conscious fear can occur when conditions are favorable, but such conscious states come about through different processes that involve different [neural] circuits. The function of the neural circuit [of fear] …is to coordinate brain and body resources to increase the chance of surviving the encounter (…)* (Ledoux, 2014).	Anger *is a syndrome of relatively specific feelings, cognitions and physiological reactions linked associatively with an urge to injure some target* (Berkowitz and Harmon-Jones, 2004).
**Responses of different physiological systems**		
(1) CNS	Fear > neutral state Amygdala, insula (Vytal and Hamann, 2010). Ventral amygdala (Whalen et al., 2001) Amygdala (Murphy et al., 2003; Phan et al., 2002). left medial temporal cortex, left hippocampal region (Strange and Dolan, 2006). Right insula (Bechara et al., 2000) Posterior insula (Rainville et al., 2006; Ekman et al., 1983). Left ACC, Cuneus (Kimbrell et al., 1999; Uchiyama, 1992). Activation of the amygdala (Adolphs et al., 1994); organized around subcortical circuits which operate preconsciously, notably the amygdala (LeDoux, 2003).	Anger > neutral state Inferior frontal Gyrus (Vytal and Hamann, 2010). Left inferior frontal gyrus, right ACC, right posterior Hypothalamus (Bechara et al., 2000). Right temporal pole, thalamus (Kimbrell et al., 1999). Left OFC, right ACC, bilateral anterior temporal poles (Dougherty et al., 1999). Increased activation of numerous cortical areas, notably the left inferior frontal gyrus (Vytal et al., 2014). Dorsal anterior cingulate cortex implied in ‘feeling angry’. Medial prefrontal cortex implied in angry rumination. Hippocampus, insula and cingulate cortex activation following provocation, predicts angry rumination (Denson et al., 2009b). ‘Basic threat system’: amygdala, hypothalamus, periaqueductal gray (Blair, 2012).
(2) ANS (physiology)	Increased respiration rate (Adolphs et al., 1994). Decreased blood supply to periphery (Rimm-Kaufman and Kagan, 1996; Robin et al., 1998; Kreibig, 2010).	Increased EMG, increased heart rate, and GSR (Ax, 1953; Schachter, 1957). Increased diastolic blood pressure (Ax, 1953; Sinha, 1996; Rochman and Diamond, 2008; Kreibig, 2010).
(3) NeuroEndocrine	Increased catecholamine levels, especially adrenaline (Frankenhaeuser, 1971; Patkai and Frankenhauser, 1965; Stemmler et al., 2001, 2007; Wacker et al., 2003).	Increased noradrenaline release (Stemmler et al., 2001, 2007; Wacker et al., 2003).
(4) Behavioral response	Withdrawal Aggression Freezing Once the stimulus is removed, the fear state dissipates	Aggression Withdrawal Persistent aggression: acting angry increases the anger state (Buschman et al.)

Four different levels of response are shown: (1) Central Nervous System (CNS); (2) Autonomous Nervous System (ANS), i.e., peripheral physiological responses; (3) endocrine; and (4) behavioral responses.

In both neuroscience and law, the classification of different subtypes of action appeals to both ‘**objective**’ and ‘**subjective**’ evidence. However, the meaning of these terms also differs between the disciplines. For neuroscience, ‘subjective’ typically refers to reports of *experiences* of action and mental states. Neuroscientific interest in subjective experience is generally based on linking specific subjective experiences with specific physiological mechanisms. Reports of subjective experience also reflect *general* cognitive biases and distortions, such as self-serving bias and confirmation bias. Neuroscientific investigations often seek to control for these wider aspects of subjective data ‘Objective’ evidence in neuroscience, instead, refers to brain events and measurements of brain function. These include neurophysiological measures (e.g., fMRI, EEG), and some implicit behavioral measures. Asking people to describe their subjective experience directly, for example, in direct reports, or through a questionnaire, would not constitute an objective measure, though their experience might also show important correlations with objective measures of brain function.

In contrast, for law, a ‘**subjective**’ element describes a behavior or situation as it was perceived by the person. An ‘**objective**’ element considers whether a **reasonable** neutral observer would have acted or perceived a situation in the same or in a similar way. There is a further ‘**subjective**’ requirement in the cases considered here: the homicidal **action** must have resulted from a loss of self-control. That is, the defendant must claim and prove that they ‘lost it’. Unfortunately, it remains unclear what amounts to a loss of self-control – there is no definition in the Coroners and Justice Act (2009). In R v Jewell, however, loss of control was defined as the ‘*loss of the ability to act in accordance with considered judgment or a loss of normal powers of reasoning*’ (R v Jewell [2014] EWCA Crim 414 [10]). One approach might involve defining loss of control by exclusion. For example, the processes of planning and deliberation that are considered necessary for murder might at first sight seem to be incompatible with the concept of loss of control. For example, one might describe pre-action conditions such as reasoning and deliberation that accompany normal human voluntary actions. Absence of these pre-action conditions woud then be necessary (but not sufficient) for demonstrating loss of control. However, this approach based on exclusion is not the path taken by the Coroners and Justice Act (2009).

Another ‘**subjective**’ requirement can be found in section 55(3). The defendant must act out of **fear** of serious violence. As with the ‘**anger**’ trigger in section 55(4) there are both ‘**subjective**’ and ‘**objective**’ requirements. The subjective requirement, for anger is that the defendant must have felt seriously wronged. The objective requirement for anger is that the feeling of being seriously wronged is justifiable. Here, ‘objective’ simply means that a person of normal tolerance and self-restraint might have reacted in the same or in a similar way to the defendant.

The “fear trigger” is considered through the lens of the person who experienced the fear response but has objective requirements. The fear reaction is assessed from the standpoint of someone of the same age and sex with a normal **degree of tolerance and self-restraint**^3^. This creates a problem for juries applying the objective test. An illustration of this is in a case where the defendant made a frenzied attack on a friend after consuming alcohol. The defendant claimed that he experienced fear of serious violence when his friend attacked him with a hammer. The appeal court decided that a jury might find when assessing the objective test that whilst the initial response might arise from fear they might conclude that the length of the attack, which was estimated at over five minutes meant that it carried on when the accused was no longer in fear *R v Goodwin* [2018] EWCA 2287.

### Aggression

Based on the neuroscientific literature, it is assumed that both fear and anger may lead to aggressive behaviors (Geen, 1990). However, these behaviors are likely to differ in important ways. The involvement of conscious awareness, and, thus, the potential for executive control, is more prominent in anger than in fear. Aggressions that happen out of fear are relatively automatic, brief, typically involve stereotyped fight or flight responses, and cease when the underlying threat ends. Typically, a fear reaction will only result in aggression if no other escape option is available: in animals flight often dominates over fight (Porges, 1995, 1997). Other studies suggest that behavioral arrest (freezing), mediated by the periaqueductal gray, occurs as a last resort only when escape (flight) and aggression (fight) possibilities are exhausted (Dhawan and Haggard, 2023). One theory (Porges, 1995, 1997) proposes that the extreme freezing response reflects a specific and ancient neural response by a dedicated brain circuit.

In contrast, anger states strongly predispose the individual to aggress. However, anger states also recruit a broader cortical network than fear (Denson et al., 2009a). They engage the amygdala projections to several cortical regions, notably the inferior frontal gyrus (IFG) (Vytal et al., 2014). The IFG is importantly involved in behavioral control, inhibition and risk aversion (Aron et al., 2003; Christopoulos et al., 2009). This link could play a key role in the capacity to check, manage or suppress anger, or at least to suppress the aggressive behaviors that it tends to cause (Berkowitz, 1993). The stop-signal reaction time task is a well-established laboratory task in which participants must stop an action they are about to make. Doing so appears to involve a specific control signal transmitted from the inferior frontal gyrus to the subcortical structures that release rapid responses and impulsive action. Lesions in the IFG area lead to difficulty in stopping a prepotent action (Aron et al., 2003; Aron and Poldrack, 2006). It seems plausible that loss of control in situations of anger could represent failures of the inferior frontal gyrus circuit to prevent subcortically-driven prepotent responses. The loss of control defense could be seen as a normative recognition that this circuit can be insufficient.

In addition, aggression out of anger is often prolonged in time, outlasting the event that triggers it, and outlasting other reactions such as fear. Angry rumination typically maintains and even auguments anger and the likelihood of aggression (Fabiansson et al., 2012). Besides, some research indicates that retaliatory aggression after a provocation is mediated by a feeling of reward, a strong motivation toward aggression, traceable also at neural level. In one fMRI study, retaliatory aggression was associated with activity in the nucleus accumbens. Further, this association was related to participants’ level of real-world violence (Chester and DeWall, 2016). Finally, in contrast to fear, aggression out of revenge-seeking appears linked to the personality traits of impulsivity and sadism (Chester and DeWall, 2018).

Proactive aggression is goal-directed and endogenous, rather than provoked by any external cue. In animals, predation is the paradigmatic form of proactive aggression. Animal research points to a second form of aggression, which can occur even without any clear threat in the environment. In mice, such spontaneous aggressive behavior is associated with activity of neurons in the ventrolateral part of the ventromedial hypothalamus (VMHvl): inactivating this region reduces spontaneous aggressive behavior (Lee et al., 2014; Falkner et al., 2016). To our knowledge, little is known about such spontaneous aggression in humans. Yet, neuroscientific evidence with humans suggest that the dorsolateral prefrontal cortex plays a crucial role in anger control and management (Denson et al., 2009b; Achterberg et al., 2016). Furthermore, lesions to these regions of the frontal cortex produce a difficulty in controlling anger (Blair, 2012).

In contrast, reactive aggression involves responding to some external cue, and comes closer to the everyday concept of evoked anger. The legal concept of ‘qualifying trigger’ clearly suggests a reactive, rather than proactive, behavior, so we focus here only on reactive aggression. In particular, we ask how neuroscientific evidence could shape our understanding of aggressive reactions to threat, and of the capacity to control such reactions.

**Figure 3:**
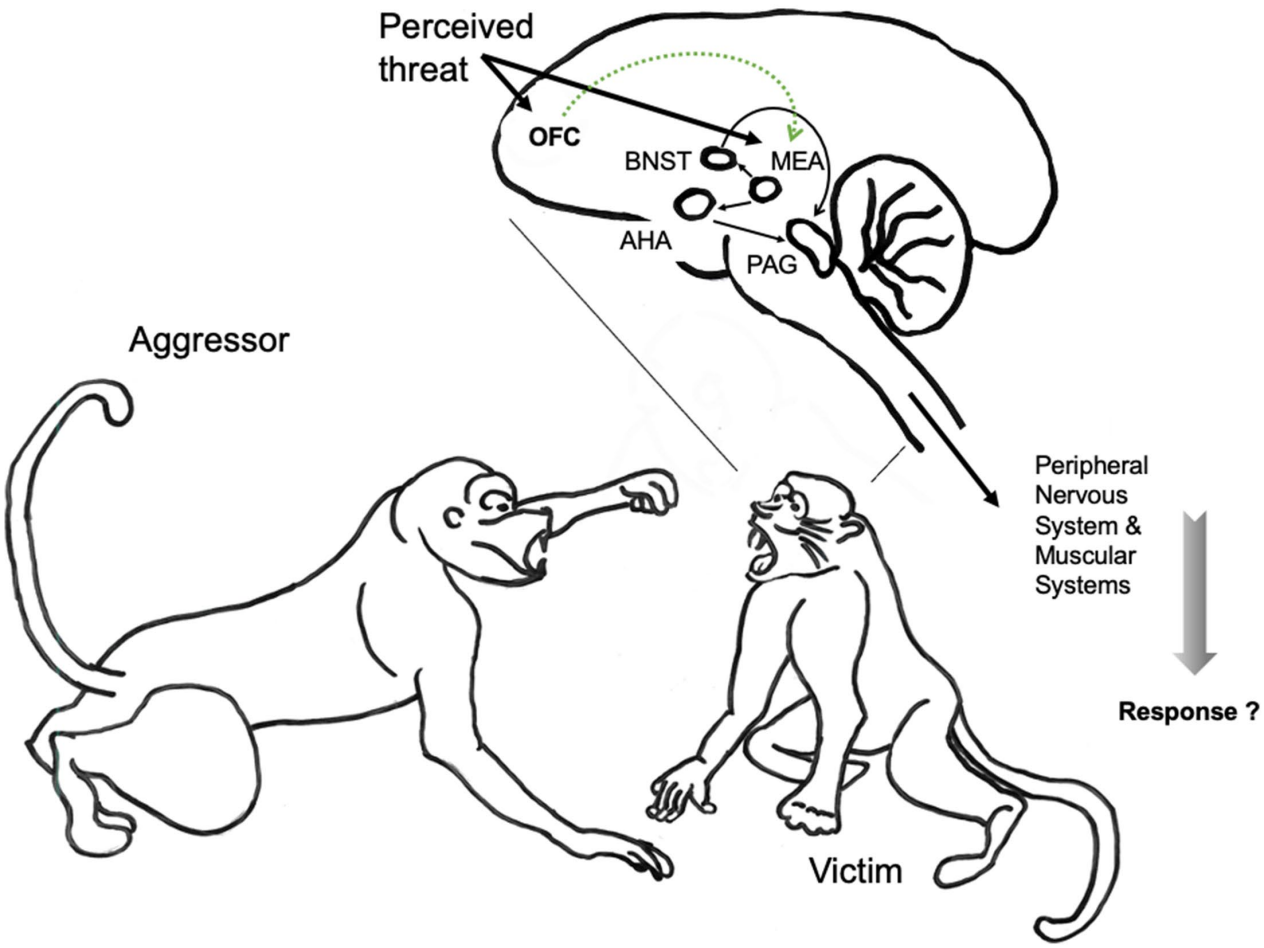
The neuroanatomical pathways of reactive aggression illustrated in the non-human primate brain (Wilson et al., 2014). Reactive aggression is often evoked by a vocal or visual signal, in this case from an aggressive conspecific. Activation of the medial amygdala (MEA) is thought to result in the activation of the bed nucleus of the stria terminalis (BNST) and the anterior hypothalamic area (AHA), which in turn activate the periaqueductal gray (PAG). In general, the orbital frontal cortex (OFC) appears to be important for the interpretation of social cues, and inhibitory inputs from the OFC might inhibit aggression by reducing responsiveness in the amygdala. Thick arrows represent inputs and outputs to and from the brain; thin arrows represent connections within the brain; dotted lines represent inhibitory connections. Illustration, text and neural models of non-human primate aggression elaborated on basis of illustration from Nelson and Trainor (2007). Drawings: © Main Author anonymized.

The neural mechanisms mediating reactive aggression out of anger are not as specific as they are for aggression out of fear, because the neural mechanisms and circuits of reactive aggression are also implicated in the regulation of other social behaviors (Newman, 1999; Nelson and Trainor, 2007). Research with rodents and non-human primates suggests that the key network underlying reactive aggression includes medial preoptic area, lateral septum, anterior hypothalamus, ventromedial hypothalamus, periaqueductal gray, medial amygdala orbitofrontal cortex and the stria terminalis. See Figure 3.

Lesions to the OFC cause impairment in the executive emotional systems, and increase reactive aggressive behavior (Damasio, 1996; Blair, 2001). Conversely, the frontal cortex inhibits reactive aggression: individuals with low basal frontal activity are more prone to aggress (Anderson et al., 1999).

## Discussion and conclusions

From a neurocognitive point of view, aggressive behavior can be associated with either anger or fear. It therefore becomes important to understand the extent to which people can control fear or anger. There is an extensive literature on therapeutic control of anxiety (Banks et al., 2007), using either reappraisal (Buhle et al., 2013) or acceptance (Hayes-Skelton et al., 2013) techniques. This literature tends to focus on therapeutic situations and on chronic anxiety, rather than on the capacity to control fear while in the situation of immediate threat. Another literature deals with fear suppression during actual threatening situations, often focussing on occupational groups such as firefighters (Smith et al., 2013). This literature suggests that preparation and training can allow individuals to continue to produce functional behaviors (e.g., fighting the fire) even under conditions of extreme threat, but does not really remove the emotion of fear or the physiological responses associated with it. Taken as a whole, this literature suggests that the physiological and behavioral responses associated with intense situational fear and threat may be difficult to control.

In contrast, aggression out of anger relies on neural mechanisms that are broadly cortical and involve association areas that are broadly identified with controlled processing. This might suggest a higher degree of voluntary control over actions during anger states than during fear states. The law specifically mentions fear and as potential qualifying triggers for a reduced responsibility for action, at least in the context of loss of control partial defense for murder (Coroners and Justice Act, 2009).

Importantly, neuroplasticity, culture and social action all allow that laws could be ‘progressive’. The law recognizes that, just as some features of our neurobiological nature make us aggressive, other features of our neurobiology also make us rational, and capable of self-control. The law expects a degree of control over behavior based on our neurobiological capacity to be rational, not just on our neurobiological capacity to be aggressive. Our investigation has one concrete implication: the law should sharply distinguish between loss of control from anger, and loss of control from fear. Anger involves a widespread neural circuit that is substantially cortical. This may explain why anger can be controlled. Fear involves specific subcortical pathways – the extent to which cortical circuits associated with volition, autonomy and rationality have control over these subcortical circuits remains a matter of research debate (Berkowitz et al., 2007). The law might reasonably require agents to suppress any tendency to aggressive acts performed in anger, while showing greater awareness of loss of control for aggressive acts performed out of fear.

## Author contributions

JC: Conceptualization, Data curation, Formal analysis, Investigation, Methodology, Validation, Visualization, Writing – original draft, Writing – review & editing. CR: Conceptualization, Data curation, Investigation, Methodology, Validation, Writing – review & editing. LC: Conceptualization, Funding acquisition, Supervision, Validation, Writing – review & editing. PH: Conceptualization, Formal analysis, Funding acquisition, Investigation, Resources, Supervision, Validation, Visualization, Writing – review & editing.

## Footnotes

^1^Words written in **bold** are terms which have important different meanings in neuroscience and law; see Figure 1 and Table 1.

^2^In addition, there are two further partial defenses to murder (diminished responsibility and suicide pact). There are also general complete defenses such as self-defense, automatism, and insanity that may lead to a full acquittal. These all lie beyond the scope of the present article.

^3^See ‘**reasonable person**’ in Table 1.
